# Allele-Specific Epigenetic Regulation of *FURIN* Expression at a Coronary Artery Disease Susceptibility Locus

**DOI:** 10.3390/cells12131681

**Published:** 2023-06-21

**Authors:** Wei Yang, Junjun Cao, David G. McVey, Shu Ye

**Affiliations:** 1 Department of Basic Medicine, Shantou University Medical College, Shantou 515041, China; 2 Department of Cardiovascular Sciences, National Institute for Health Research, Leicester Biomedical Research Centre, University of Leicester, Leicester LE3 9QP, UK; 3 Cardiovascular-Metabolic Disease Translational Research Programme, Yong Loo Lin School of Medicine, National University of Singapore, Singapore 117597, Singapore

**Keywords:** genetic variant, genetic association, epigenetics, DNA methylation, *FURIN*, MeCP2

## Abstract

Genome-wide association studies have revealed an association between the genetic variant rs17514846 in the *FURIN* gene and coronary artery disease. We investigated the mechanism through which rs17514846 modulates *FURIN* expression. An analysis of isogenic monocytic cell lines showed that the cells of the rs17514846 A/A genotype expressed higher levels of *FURIN* than cells of the C/C genotype. Pyrosequencing showed that the cytosine (in a CpG motif) at the rs17514846 position on the C allele was methylated. Treatment with the DNA methylation inhibitor 5-aza-2′-deoxycytidine increased *FURIN* expression. An electrophoretic mobility super-shift assay with a probe corresponding to the DNA sequence at and around the rs17514846 position of the C allele detected DNA-protein complex bands that were altered by an anti-MeCP2 antibody. A chromatin immunoprecipitation assay with the anti-MeCP2 antibody showed an enrichment of the DNA sequence containing the rs17514846 site. siRNA-mediated knockdown of MeCP2 caused an increase in FURIN expression. Furthermore, MeCP2 knockdown increased monocyte migration and proliferation, and this effect was diminished by a FURIN inhibitor. The results of our study suggest that DNA methylation inhibits *FURIN* expression and that the coronary artery disease-predisposing variant rs17514846 modulates *FURIN* expression and monocyte migration via an allele-specific effect on DNA methylation.

## 1. Introduction

Genome-wide association studies have revealed a robust association between genetic variation on chromosome 15q26.1 and coronary artery disease (CAD) susceptibility [1,2,3,4]. The A allele of the lead single nucleotide polymorphism (SNP), rs17514846, at this locus is associated with increased CAD risk [1,2,3,4]. rs17514846 is located in a noncoding region of the *FURIN* (FES Upstream Region) gene and modulates monocyte FURIN expression [5]. The *FURIN* gene encodes the subtilisin-like proprotein convertase FURIN that possesses proteolytic activity to cleave the prodomain off of, and thereby activate, many proteins. Reported substrates of FURIN include PCSK9 (proprotein convertase subtilisin/kexin type 9) [6], MMP14 (matrix metalloproteinase 14) [7], TGFβ1 (transforming growth factor beta 1) [8], IGF1 (insulin-like growth factor 1) [9], NGF (nerve growth factor) [10], and integrin-α(v) [11], as well as several members of the ADAM (a disintegrin and metalloproteinase domain-containing protein) and ADAMTS (a disintegrin and metalloproteinase with thrombospondin motifs) protein families, including ADAM10 [12], ADAM17 [13], DAMTS4 [14], and ADAMTS7 [15].

There is accumulating evidence indicating that FURIN plays an important role in atherosclerosis, the pathological condition underlying CAD. Studies have shown increased expression of FURIN in human atherosclerotic plaques and in rabbit and rat arterial neointimal lesions induced by vascular injury [16,17,18]. In atherosclerotic plaques, FURIN has been detected in macrophages, smooth muscle cells, and endothelial cells [16,19].A key process in the development and progression of atherosclerosis is the recruitment of monocytes into the arterial wall and atherosclerotic lesions [20]. Our recent study showed that FURIN promotes human monocyte migration and proliferation [5]. In agreement, a mouse model study by other investigators demonstrated that FURIN inhibition reduces monocyte migration and retards atherosclerotic lesion progression [21]. Furthermore, a clinical study reported an association between high FURIN levels and increased recurrent cardiovascular events and mortality in patients with acute myocardial infarction [22].

It is currently unknown how the CAD-associated SNP rs17514846 modulates FURIN expression. Our present study sought to investigate the underlying regulatory mechanism. The study finds that the non-risk allele (the C allele) is subjected to methylation of a CpG motif at the rs17514846 site, interacts with the transcription factor MeCP2 (that often acts as a gene repressor by binding to methylated CpG sites [23,24]), and results in reduced expression of FURIN, whereas the C to A change of this SNP in the CAD risk allele (the A allele) abolishes the CpG motif, diminishes MeCP2 binding, and leads to increased FURIN expression. Furthermore, our study shows that targeted knockdown of MeCP2 results in an increase in FURIN expression and promotes monocyte migration and proliferation, whereas treatment with a FURIN inhibitor reduces the effect of MeCP2 on monocyte migration and proliferation.

## 2. Materials and Methods

### 2.1. Generation of Isogenic Cell Lines

THP1 monocytes (ATCC, TIB-202) were used as parental cells to generate isogenic cell lines with A/A or C/C genotypes at the rs17514846 position with CRISPR (clustered regularly interspaced short palindromic repeats) [5]. In brief, two gRNAs (guideRNA; 5′-GTCTGTGGGGGTCTCATTTTC-3′ and 5′-GCCCACATCCTCTGTTAAATG-3′, respectively) were separately cloned into a gRNA/Cas9 expression vector containing a green fluorescent protein reporter (p-UG-gRNA-EFS-Cas9-T2A-EGFP-WPRE). Additionally, a homology-directed repair donor vector was prepared by inserting 5′ and 3′ homology aims at either side of the to-be-edited sites and subsequently subjected to site-directed mutagenesis to generate a vector containing the A allele at rs17514846. A PGK-Neo-PolyA cassette was also inserted in between the 5′ and 3′ arms of these vectors to provide a means for the selection of edited THP-1 cells after transduction. THP1 cells, homozygous for the C allele of rs17514846, were transduced with the lentivirus particles containing the gRNAs and the A allele donor vector and incubated for 96 h, followed by fluorescence-activated cell sorting. Sorted cells were cultured under G418 selection to select successfully edited cells. The selected cells were screened via polymerase chain reaction (PCR) and Sanger sequencing to confirm the successful editing of rs17514846 from the parental C/C genotype to A/A. Isogenic THP-1 cells (containing the G418 cassette) of the C/C genotype and isogenic THP-1 cells (containing the G418 cassette) of the A/A genotype were subsequently subjected to quantitative reverse transcriptase polymerase chain reaction assays of the FURIN gene.

### 2.2. Cell Culture and 5-Aza-2-Deoxycytidine Treatment

THP1 cells and the isogenic cell lines described above were cultured in RPMI 1640 medium supplemented with 10% fetal bovine serum and 1% penicillin-streptomycin at 37 °C with 5% CO_2_. In some experiments, cells were first incubated with either 10 µM 5-aza-2′-deoxycytidine (dissolved in dimethylsulfoxide) or the solvent alone for 72 h.

### 2.3. Quantitative Reverse Transcriptase Polymerase Chain Reaction

Total RNA from isogenic THP1 cells was extracted with the use of Trizol reagent (Takara, 9109), reverse-transcribed into cDNA with a PrimeScript RT Reagent Kit (Takara, RR047A), and subjected to quantitative real-time polymerase chain reaction with a TB Green Premix Ex Taq II kit (Takara, RR820A). The PCR program consisted of 95 °C for 1 min, and then 40 cycles of 95 °C for 15 s, 60 °C for 30 s, and 72 °C for 30 s. The PCR primers were designed with Primer-BLAST [25] (https://www.ncbi.nlm.nih.gov/tools/primer-blast/index.cgi?LINK_LOC=BlastHome, accessed on 8 June 2023). The specificity of primers was checked with the BLAST function of Primer-BLAST [25], confirming that the primers were expected to amplify only the intended region of the *FURIN* gene and nowhere else in the genome. The sequences of the primers for amplifying *FURIN* transcript isoforms 1 and 2 (NM_002569.4 and NM_001382622.1), *FURIN* transcript isoform 3 (NM_001289823.2), *FURIN* transcript isoform 4(NM_001289824.2), an exon shared by multiple different *FURIN* isoforms, and the reference housekeeping gene *ACTB*, respectively, are shown in Supplementary Table S1. *FURIN* expression levels were calculated with the use of the 2^−∆∆Ct^ method.

### 2.4. Pyrosequencing

Genomic DNA was subjected to bisulfite modification and purification with the EpiJET bisulfite conversion kit (ThermoFisher Scientific, Vilnius, Lithuania, K1461), according to the manufacturer’s protocol. Briefly, 500 ng of DNA in 20 µL was added to a 120 µL modification reagent solution, mixed thoroughly, and incubated at 98 °C for 10 min and then at 60 °C for 4 h. The modified DNA was purified, subjected to de-sulfonation at room temperature for 20 min, washed, and dissolved in 40 µL elution buffer. The recovered DNA was used as a template in a methylation-specific polymerase chain reaction (PCR) to amplify the target DNA segment with biotin-labeled methylation-specific primers designed with Pyromark Assay Design Software 2.0 (Qiagen, Singapore). The primer sequences are shown in Supplemental Table S1. The PCR products were mixed with magnetic beads coated with streptavidin and isolated with a Pyromark Q24 Vacuum Workstation (Qiagen), followed by pyrosequencing with a PyroMark Q24 instrument (Qiagen). The sequencing signals were analyzed with Pyromark Q24 2.0.6 software (Qiagen).

### 2.5. Bioinformatics Analysis

PROMO [26,27] (https://alggen.lsi.upc.es/cgi-bin/promo_v3/promo/promoinit.cgi?dirDB=TF_8.3, accessed on 15 December 2022) and TRANSFAC (http://gene-regulation.com/pub/databases.html, accessed on 10 February 2023) were used tosearch for transcription factor binding motifs in the DNA sequence at and surrounding the rs17514846 site. Subsequently, the implicated transcription factor was searched in the JASPAR [28] (https://jaspar.genereg.net/, accessed on 10 February 2023) and Cistrom DB [29,30] (http://cistrome.org/db/#/, accessed on 10 February 2023) databases to further check that its binding motif matched the sequence containing the rs17514846 site.

### 2.6. Electrophoretic Mobility Super-Shift Assay

Nuclear proteins were extracted from THP1 cells with NE-PER Nuclear and Cytoplasmic Extraction Reagents (ThermoFisher, Vilnius, Lithuania, 78833). Biotin-labeled double-stranded oligonucleotide probes corresponding to the DNA sequences containing and surrounding the rs17514846 site (5′-AGTTGCGCCTGA[C/A]GCCTGCTTTCTT-3′), of either the C or A allele, were individually incubated with or without nuclear protein extracts, in the presence or absence of an unlabeled C allele probe, an unlabeled A allele probe, or an unlabeled non-specific oligonucleotide, for 20 min at 4 °C. For the super-shift assay, the nuclear protein extracts were first incubated with a rabbit anti-human MeCP2 antibody or an equivalent amount of isotype rabbit IgG antibody for 30 min at 4 °C, followed by incubation with the biotin-labeled C allele probe for 20 min at 4 °C. The mixes were electrophoresed on a 6.5% non-denaturing polyacrylamide gel and subsequently transferred onto a nylon membrane. The membrane was incubated with streptavidin-conjugated horseradish peroxidase and then with reagents of the Chemiluminescent Nucleic Acid Detection Module Kit (ThermoFisher, Vilnius, Lithuania, 89880). The membrane was then scanned with the ChemiDoc XRS+ system (Bio-Rad Laboratories, Hercules, CA, USA).

### 2.7. Chromatin Immunoprecipitation and qPCR

Chromatin immunoprecipitation was performed with the use of a ChIP kit (Abcam, Cambridge, UK, ab270816). Briefly, THP1 cells were treated with 1% formaldehyde for 10 min to induce protein crosslinking. The crosslinking reaction was terminated by adding glycine. Thereafter, the cells were washed with ice-cold phosphate buffered saline and then lysed on ice with the lysis buffers of the ChIP kit. The chromatin DNA was sheared into fragments of between 200 and 1200 base pairs in length by sonication (Covaris, Brighton, UK). The chromatin DNA-protein complexes were incubated with an anti-MeCP2 antibody (Cell Signaling Technology, Danvers, MA, USA, 3456) or an isotype control antibody (Abcam, ab37415) at 4 °C overnight. The immunocomplexes were precipitated with protein-A-coated magnetic beads. DNA in the immunocomplexes was then isolated and subjected to a quantitative PCR analysis of the FURIN gene, standardized against the input DNA sample, using the 2^−∆∆Ct^ method. The sequences of the PCR primers are shown in Supplemental Table S1. The results are expressed as the fold difference by comparing the quantitative PCR values from the samples pulled down by the anti-MeCP2 antibody versus the quantitative PCR values from the samples pulled down by the isotype control antibody. Quantitative PCR with a human negative control primer set (obtained from Active Motif, catalog number 71001) was also performed to serve as a negative control for the chromatin immunoprecipitation analysis.

### 2.8. Transfection of Small Interference RNA

Small interference RNA (siRNA) targeting *MeCP2* and a negative control siRNA were synthesized by GenePharma (Shanghai, China). The sequences of the *MeCP2* siRNA were 5′-GGAAAGGACUGACCUGUUUU-3′ and 5′-AACAGGUCUUCAGUCCUUUCCUU-3′. The sequences of the negative control siRNA were 5′-UUCUCCGAACGUGUCACGUTT-3′ and 5′-ACGUGACACGUUCGGAGAATT-3′. THP1 cells were transfected with either the MeCP2 siRNA or control siRNA with the use of Lipofectamine RNAiMAX Transfection Reagent (Invitrogen, Waltham, MA, USA, 13778) and subjected to downstream experiments at 48 h or 72 h post-transfection.

### 2.9. Western Blotting Analysis

Protein extracts were prepared from THP1 cells and isogenic cells of the rs17514846 C/C and A/A genotypes, respectively. The protein extracts were subjected to denaturing polyacrylamide gel electrophoresis and then transferred onto a polyvinylidene difluoride membrane. The membrane was incubated with either a rabbit anti-FURIN antibody (Abcam, ab183495), a rabbit anti-MeCP2 antibody (Cell Signaling Technology, Danvers, MA, USA, 3456), or a rabbit anti-β-actin antibody (Sangon Biotech, Shanghai, China, D110001) at 4 °C overnight, washed, and then incubated with a fluorescein-labeled goat anti-rabbit secondary antibody (LI-COR Biosciences, 926-32211). The protein bands were imaged with the ChemiDoc XRS+ System (Bio-Rad) and analyzed with ImageJ software (1.4.3.67).

### 2.10. Cell Migration Assay

THP1 cells were transfected with either the MeCP2 siRNA or negative control siRNA for 48 h and then incubated with 2.5 µM CellTracker Green CMFDA (5-chloromethylfluorescein diacetate) (Invitrogen, C7025) for 30 min. Thereafter, cells were washed, suspended in RPMI 1640 medium without serum, and seeded into the upper chamber of trans-wells on a 96-well plate with 7 × 10^4^ cells per well. RPMI 1640 medium complemented with 10% fetal calf serum was added into the lower chamber of each well. In some assays, the FURIN inhibitor decanoyl-RVKR-CMK (GLPBIO, GC15108) was additionally added to both the upper and lower chambers at a final concentration of 10 µM. Cells were then incubated in a 5% CO_2_ atmosphere at 37 °C for 24 h. Cells that had migrated onto the lower chamber were observed under a fluorescence microscope, and cells in seven randomly selected areas were photographed and counted. The average number of cells in the seven areas is presented.

### 2.11. Cell Proliferation Assay

THP1 cells were transfected with either the *MeCP2* siRNA or the negative control siRNA for 48 h and then seeded on a 96-well plate with 1 × 10^4^ cells per well. In some assays, cells were incubated with the FURIN inhibitor decanoyl-RVKR-CMK (GLPBIO; GC15108) at a final concentration of 10 µM for 24 h. Cells were then subjected to a proliferation assay with the Cell Counting Kit-8 (MedChemExpress, HY-K0301).

### 2.12. Cell Apoptosis Assay

THP1 cells were transfected with either the *MeCP2* siRNA or the negative control siRNA for 48 h and then seeded on a 6-well plate with 3.5 105 cells per well. In some assays, cells were incubated with the FURIN inhibitor decanoyl-RVKR-CMK (GLPBIO, GC15108) at a final concentration of 10 µM for 24 h. Cells were then subjected to an apoptosis assay with the use of FITC Annexin V Apoptosis Detection Kit I (BD Pharmingen, 556547).

### 2.13. Statistical Analyses

A Mann-Whitney test was used to ascertain differences between experimental groups in the *FURIN* expression level, fold enrichment of DNA in the chromatin immunoprecipitation-qPCR analysis, standardized Western blotting band intensity, and the rates of cell migration, proliferation, and apoptosis, respectively. A *p* < 0.05 in a 2-tailed test was considered statistically significant.

## 3. Results

### 3.1. Allelic Effect of rs17514846 on FURIN Expression

**Figure 1:**
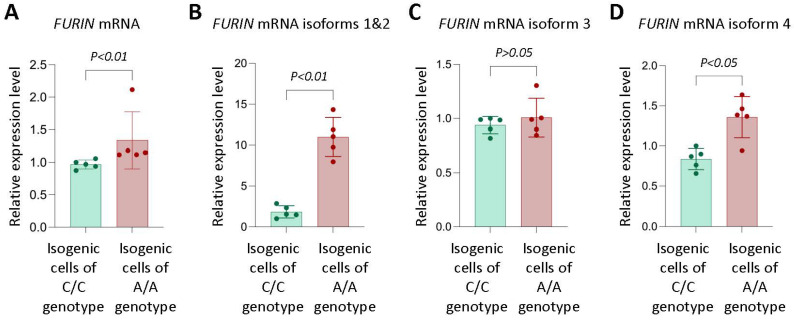
Monocytic cells of rs17514846 A/A genotype express higher levels of FURIN. than monocytic cells of the C/C genotype. Results of qRT-PCR analysis of FURIN in isogenic monocytic cells of either the rs17514846 C/C or A/A genotype. Data shown are mean (±standard deviation) of FURIN relative expression levels standardized against the expression levels of the reference housekeeping gene ACTB; *p* values from Mann-Whitney test; *n* = 5 in each group.

To investigate the influence of rs17514846 on *FURIN* expression level, we performed a quantitative reverse transcriptase polymorphism chain reaction (qRT-PCR) analysis of the *FURIN* transcript isoforms [31] in two isogenic cell lines (derived from THP1 monocytic cells) that were genetically identical, apart from the rs17514846 position, where one of these isogenic lines was homozygous for the C allele while the other isogenic line was homozygous for the A allele. The analysis showed that isogenic cells of the rs17514846 A allele had higher expression of *FURIN*, particularly transcript isoforms 1 and 2 (NM_002569.4 and NM_001382622.1), and isoform 4 (NM_001289824.2), than isogenic cells of the C allele (Figure 1).

### 3.2. Allelic Effect of rs17514846 on DNA Methylation

**Figure 2:**
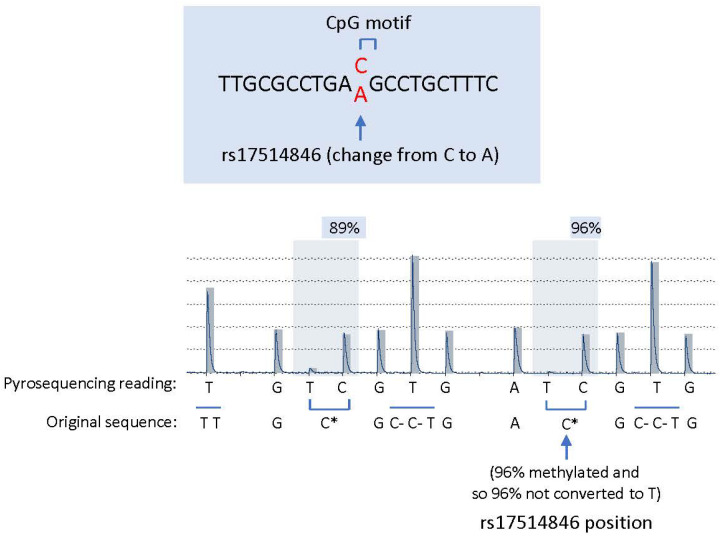
rs17514846 abolishes a methylated CpG motif.Upper panel: the rs17514846 resides in a CpG site, and the C to A change abolishes this CpG site, namely, the C allele (TTGCGCCTGACGCCTGCTTTC), but not the A allele (TTGCGCCTGAAGCCTGCTTTC), which contains this CpG motif. Lower panel: a pyrosequencing methylation analysis of THP1 monocytic cells showed that in the rs17514846 C allele, the cytosine in this CpG motif was methylated. C* denotes methylated cytosine that was not converted to T in pyrosequencing; C- indicates unmethylated cytosine that was converted to T in pyrosequencing.

The rs17514846 SNP resides in a CpG site, and the C to A change abolishes this CpG site, namely, the C allele (TTGCGCCTGACGCCTGCTTTC), but not the A allele (TTGCGCCTGAAGCCTGCTTTC), comprises part of the CpG motif. Therefore, we wondered whether this CpG site was subjected to methylation. A pyrosequencing methylation analysis of THP1 monocytic cells (which were of the rs17514846 C/C genotype) showed a high level of 5-methylcytosine (5-mC) at this CpG site (Figure 2). Several other CpG motifs nearby were also found to be methylated (Figure 2).

### 3.3. DNA Methylation Inhibition Increased FURIN Expression

**Figure 3:**
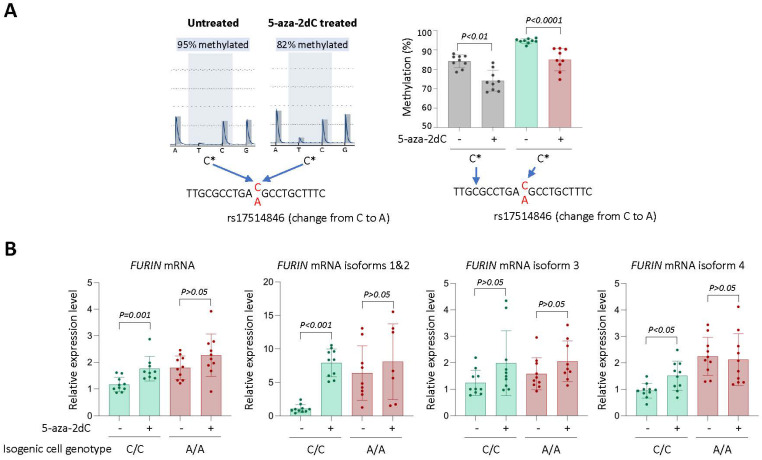
DNA methylation inhibition increases *FURIN* expression in isogenic monocytic cells of the rs17514846 C/C genotype. (**A**). Pyrosequencing analysis of THP1 monocytic cells (of the rs17514846 C/C genotype) treated with the DNA methylation inhibitor 5-aza-2-deoxycytidine (5-aza-2dC, 10 µM) for 24 h. Left: average representative programs at the rs17514846 position; right: mean percentage (±standard deviation) of methylated cytosine (C*) at the respective indicated position; *p* values from Mann-Whitney test; *n* = 9 in each group. (**B**). *FURIN* RT-PCR analysis of isogenic monocytic cells of either the rs17514846 C/C or A/A genotype, treated with 5-aza-2dC (10 µM) for 72 h. Data shown are mean (±standard deviation) of *FURIN* relative expression levels standardized against the expression levels of the reference housekeeping gene *ACTB*; *p* values from Mann-Whitney test; *n* = 7–10 in each group.

Since rs17514846 resides in a methylated CpG site, we sought to investigate if DNA methylation at this site influenced *FURIN* expression. We found that incubating THP1 monocytic cells with a DNA methylation inhibitor, 5-aza-2′-deoxycytidine, attenuated methylation of the cytosine at the rs17514846 site (Figure 3A). Furthermore, a qRT-PCR analysis showed that treatment with this DNA methylation inhibitor increased *FURIN* expression in isogenic cells of the rs17514846 C/C genotype, particularly *FURIN* transcript isoforms 1 and 2 and isoform 4 (Figure 3B). In contrast, in isogenic cells of the A/A genotype, there were no significant changes in *FURIN* expression following treatment with the DNA methylation inhibitor (Figure 3B).

### 3.4. Allele-Differential Binding of the Transcription Factor MeCP2

Since the rs17514846 CpG site in the C allele is methylated and this CpG motif is abolished in the A allele, we wondered whether this would affect transcription factor binding to the DNA in this region. A bioinformatic analysis indicated a potential interaction of the rs17514846 site with the transcription factor MeCP2, which often functions as a gene repressor by binding to DNA with methylated CpG sites [23,24].

**Figure 4:**
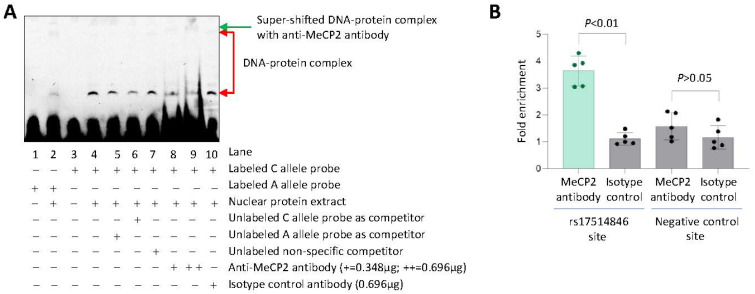
Preferential binding of MeCP2 to the rs17514846 C allele. (**A**). An average representative image of electrophoretic mobility super-shift assay with probes corresponding to either the rs17514846 C or A allele, protein extracts from THP1 monocytic cells, an anti-MeCP2 antibody, and an isotype control antibody (**B**). Results of chromatin immunoprecipitation analysis of MeCP2 in THP1 cells. Data shown are mean (±standard deviation) of fold enrichment of the respective DNA sequence in anti-MeCP2 antibody immunoprecipitated DNA sample as compared to that of isotype control IgG precipitated sample, as determined by quantitative PCR analysis. *p* values are from Mann-Whitney test; *n* = 5 in each group.

In support of this hypothesis, an electrophoretic mobility super-shift assay using a probe corresponding to the DNA sequence at and surrounding rs17514846 with the C allele, together with THP1 monocytic cell nuclear protein extracts, detected two DNA-protein complex bands (Figure 4A, lane 4), possibly indicating post-translational modification of the protein in one of these bands. Adding an anti-MeCP2 antibody to the assay resulted in a reduction in the intensity of one of these bands (Figure 4A, lanes 8 and 9, lower band) and a super-shift of the other band (Figure 4A, lanes 8 and 9, upper band), indicating binding of MeCP2 to the rs17514846 site of the C allele. In contrast, the DNA-protein complex bands were barely detectable using a probe corresponding to the A allele (Figure 4A, lane 2).

In further support, a chromatin immunoprecipitation assay of THP1 monocytic cells (which were of the rs17514846 C/C genotype) detected an enrichment of the DNA sequence containing the rs17514846 site in chromatin precipitates pulled down by an anti-MeCP2 antibody, compared with chromatin precipitates pulled down with an isotype control antibody (Figure 4B).

### 3.5. Knockdown of MeCP2 Increased FURIN Expression

**Figure 5:**
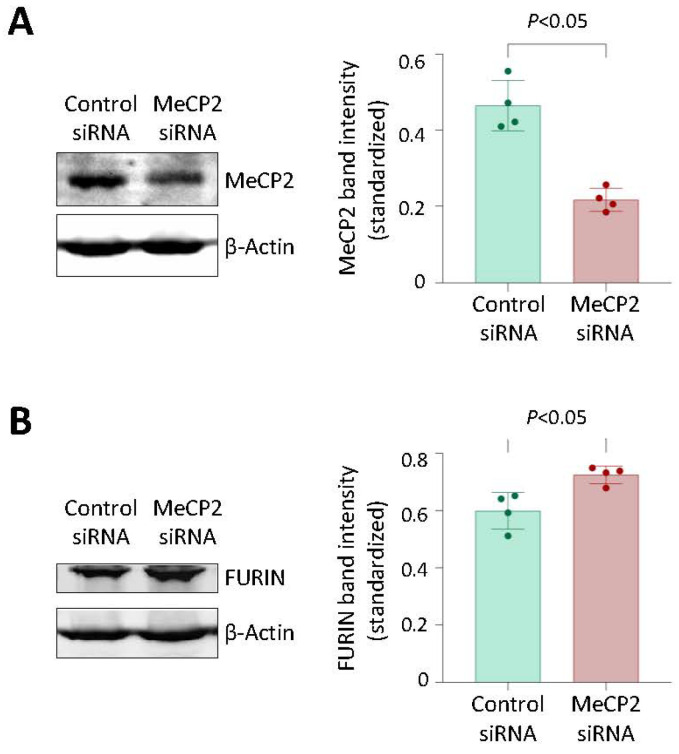
MeCP2 knockdown increases FURIN expression. Results of Western blotting analyses of MeCP2 (**A**) and FURIN (**B**) in THP1 monocytic cells transfected with either a *MeCP2* siRNA or negative control siRNA for 72 h. **Left**: average representative images of Western blotting images; **right**: mean (±standard deviation) of MeCP2 band intensity (**A**) and FURIN band intensity (**B**), standardized against band intensity of the reference housekeeping protein β-actin. *p* values are from Mann-Whitney test; *n* = 4 in each group.

Since MeCP2 often functions as a gene transcription repressor [23,24], we investigated if the knockdown of MeCP2 in cells would alter the FURIN expression level. The analysis showed that siRNA-mediated knockdown of MeCP2 increased FURIN expression in THP1 monocytic cells (which were of the rs17514846 C/C genotype) (Figure 5), suggesting that MeCP2 inhibits FURIN expression.

### 3.6. MeCP2 Knockdown Increased Monocyte Migration and Proliferation, and These Effects were Attenuated by a FURIN Inhibitor

**Figure 6:**
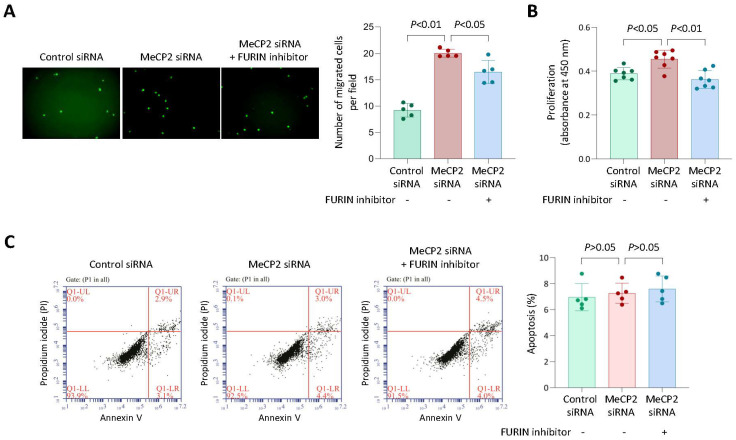
MeCP2 knockdown increases monocyte proliferation and migration, and these effects are attenuated by a FURIN inhibitor. Results of migration (**A**), proliferation (**B**), and apoptosis (**C**) assays of THP1 monocytic cells transfected with either a *MeCP2* siRNA or negative control siRNA for 48 h, with or without treatment with the FURIN inhibitor decanoyl-RVKR-CMK for 24 h. Data shown are mean (±standard deviation) values and *p* values from Mann-Whitney test; *n* = 5 (**A**,**C**) or *n* = 7 (**B**) in each group.

Previous studies have shown that FURIN promotes monocyte migration and proliferation [5,21]. Therefore, we investigated the possibility that the inhibitory effect of MeCP2 on FURIN expression has a bearing on monocyte migration and proliferation. We found that MeCP2 knockdown in THP1 monocytic cells increased their migration and proliferation, and this effect was diminished by a FURIN inhibitor (decanoyl-RVKR-CMK) (Figure 6A,B), suggesting that inhibition of FURIN expression by MeCP2 reduces monocyte migration and proliferation. MeCP2 knockdown did not affect the rate of monocyte apoptosis (Figure 6C).

## 4. Discussion

Genome-wide association studies have identified many genomic loci associated with CAD susceptibility [32,33]. However, the underlying biological mechanisms are still unclear for the majority of these loci, which hinders the translation of genetic discoveries into new treatments. Many of the identified CAD susceptibility loci are not associated with the classic risk factors such as hypercholesterolemia and hypertension [1,34,35], suggesting that they do not act through the traditional pathways and are thus not addressed by current treatments, which primarily focus on lowering cholesterol levels and controlling blood pressure. Hence, there is a need to identify novel therapeutic targets for the development of new treatments that complement current strategies that target conventional risk factors.

rs17514846 has been reported as the lead CAD-associated SNP at the 15q26.1 locus in genome-wide association studies [1], and this variant has been shown to influence FURIN expression in monocytes [5]. Our present study reveals a mechanism through which rs17514846 modulates FURIN expression. Our study shows that rs17514846 resides in a CpG motif in the presence of the C allele, which is methylated in monocytes. The C-to-A substitution producing the A allele of rs17514846 abolishes this methylated CpG motif. Our study reveals that the C allele interacts with MeCP2, a transcription factor known to be capable of binding to methylated DNA and acting as a transcription repressor [23,24]. Furthermore, our study demonstrates that inhibition of DNA methylation with the use of 5-aza-2′-deoxycytidine or knockdown of MeCP2 can increase FURIN expression in monocytes. Taken together, these results suggest that the C-to-A substitution of rs17514846 abolishes DNA methylation at this site and thereby diminishes MeCP2 binding to this region, consequently leading to increased FURIN expression.

Previous studies have shown that FURIN expression is under the regulation of several transcription factors interacting with their respective binding sites in the FURIN regulatory regions, including C/EBP-β (CCAAT enhancer binding protein β) [31], HIF1 (hypoxia-inducible actor-1) [36], GATA1 (GATA binding protein 1) [37], SMAD2/4 (SMAD family member 2 and SMAD family member 4) [38], and STAT4 (signal transducer and activator of transcription 4) [39]. Our present study uncovers that FURIN expression is also regulated by the transcription factor MeCP2, which binds to the rs17514846 site in intron 1 of the *FURIN* gene. Whereas C/EBP-β, HIF1, GATA1, SMAD2/4, and STAT4 enhance FURIN expression as shown in previous studies [31,36,37,38,39], MeCP2 displayed an inhibitory effect on FURIN expression in our study. Taken together, the findings from the previously reported studies [31,36,37,38,39] and our present work indicate that FURIN expression is subjected to both positive and negative regulation.

FURIN is a potential therapeutic target associated with many diseases, including atherosclerosis [21] and cancer [40], and therefore there has been considerable research into the development of FURIN inhibitors [41]. The findings of our present study indicate a possible new approach for targeting FURIN, namely, inhibiting FURIN expression by inducing/increasing DNA methylation in the FURIN gene. There are emerging methodologies that can potentially be used to achieve this. For example, it has been demonstrated that an inactive Cas9 fused with an engineered DNA methyltransferase can induce locus-specific DNA methylation without affecting global methylation [42]. Thus, controlling FURIN expression by inducing targeted DNA methylation offers another strategy for FURIN targeting that warrants further investigation.

Our study demonstrates that a genetic variant can modulate gene expression through an epigenetic mechanism. As such a mechanism might operate at other loci as well, a possible genetic-epigenetic interaction would be worth considering in mechanistic studies of other disease-associated loci.

In summary, our study shows that: (1) the CAD-predisposing genetic variant rs17514846 abolishes a methylated CpG motif in the *FURIN* gene; (2) the rs17514846 C allele interacts with the transcription factor MeCP2, which is known to often function as a gene repressor by binding to methylated CpG sites; (3) treatment with a DNA methylation inhibitor increases FURIN expression; (4) MeCP2 attenuation increases FURIN expression; (5) MeCP2 attenuation increases monocyte migration and proliferation, whereas a FURIN inhibitor diminishes this effect.

Thus, the results of our study provide a novel mechanistic understanding of the effect of the CAD-associated variant rs17514846 on FURIN expression and monocyte migration/proliferation. The finding that DNA methylation suppresses *FURIN* expression suggests that inducing targeted DNA methylation at the *FURIN* gene could be considered as a possible approach for FURIN inhibition in drug development for CAD intervention.

## Data Availability

Data is contained within the article or Supplementary Materials.

## Supplementary Materials

The following supporting information can be downloaded at: https://www.mdpi.com/article/10.3390/cells12131681/s1, Table S1: Primer sequences for qPCR, methylation-specific PCR & pyrosequencing ChIP-qPCR.
