# Supplementary material for: Allele-Specific Epigenetic Regulation of FURIN Expression at a Coronary Artery Disease Susceptibility Locus
Source: Cells. 2023 Jun 21;12(13):1681. doi: 10.3390/cells12131681 (PMC10341058; doi:10.3390/cells12131681)
Supplement: Supplementary file 1 [file cells-12-01681-s001.zip › cells-2436738-supplementary.pdf]

Supplementary Table S1

***FURIN* mRNA**	
PCR forward primer	CCAGGCCACATGACTACTCC
PCR reverse primer	CTTGGTCAGCGTCCCATAGT
***FURIN* transcript isoforms 1&2**	
PCR forward primer	AGGGATAGGAGCCTGACTGTT
PCR reverse primer	AAGAGTGCCGACCTTCACC
***FURIN* transcript isoform 3**	
PCR forward primer	GCTCCCCAGGGGTCG
PCR reverse primer	GCTGCTACCACCCATAGCAA
***FURIN* transcript isoform 4**	
PCR forward primer	GCTCTCTGTGTCAGGAGGTTCT
PCR reverse primer	CCCTCTAGTCCCAGTTTGTCTC
ACTB	
PCR forward primer	CTGGAACGGTGAAGGTGACA
PCR reverse primer	AAGGGACTTCCTGTAACAATGCA
**Methylation-specific PCR and pyrosequencing**	
PCR forward primer	TATGGTTGGGGTTGTTGGAT
PCR reverse primer	Bio-CCAACTATTTAATTACCTTACAACACT
Sequencing primer	TTTTTTTGGGGGGAT
**ChIP-qPCR**	
PCR forward primer	GGCTGGGGTTGTTGGATAGA
PCR reverse primer	GGAAAGAAAGCAGGCGTCAG
